# Biobased Imine Vitrimers Obtained by Photo and Thermal Curing Procedures—Promising Materials for 3D Printing

**DOI:** 10.1021/acsapm.3c03234

**Published:** 2024-03-14

**Authors:** Anna Vilanova-Pérez, Silvia De la Flor, Xavier Fernández-Francos, Àngels Serra, Adrià Roig

**Affiliations:** † Department of Analytical and Organic Chemistry, Universitat Rovira i Virgili, C/Marcel·lí Domingo 1, Edif. N4, 43007 Tarragona, Spain; ‡ Department of Mechanical Engineering, Universitat Rovira i Virgili, Av. Països Catalans 26, 43007 Tarragona, Spain; § Thermodynamics Laboratory ETSEIB, Universitat Politècnica de Catalunya, Av. Diagonal 647, 08028 Barcelona, Spain

**Keywords:** vitrimer, imine metathesis, 3D printing, vanillin, methacrylate

## Abstract

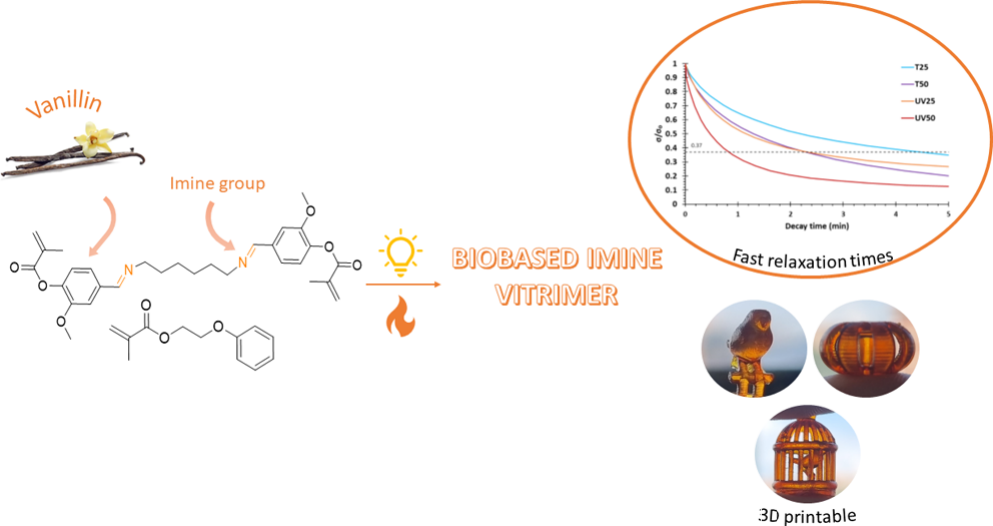

Imine-based vitrimers were prepared from synthesized diimine-dimethacrylate monomer derived from biobased vanillin. First, a methacrylate derivative starting from vanillin was synthesized. The diimine derivative was synthesized by condensation of the aldehyde groups from two vanillin methacrylate units with the amine groups of hexamethylenediamine (HMDA). The synthesized product was used in formulations containing ethylene glycol phenyl ether methacrylate (EGPMA) as a reactive diluent for the customization of final material properties and cured by exposure to ultraviolet (UV)-light using suitable radical photoinitiators or else with temperature using a radical thermal initiator. Materials with glass transition temperatures (*T*_g_s) ranging from 70 to 90 °C were prepared, showing good thermal stability and mechanical and thermomechanical properties. The evaluation of their vitrimeric characteristics revealed that all materials achieved a stress-relaxation factor (σ = 0.37σ_0_) in less than 130 s at 160 °C, with photocured materials exhibiting faster relaxation rates. The catalytic effect of phosphine oxide groups in imine metathesis has also been evidenced. All prepared materials could be mechanically recycled and completely solubilized in a two-step degradation process, putting evidence of their potential use for carbon fiber-reinforced composites (CFRCs). In addition, they demonstrated promising self-repairing abilities. Finally, as a proof of concept, it was established that these formulations could be effectively processed using a Digital Light Processing three-dimensional (3D) Printer (DLP), resulting in the fabrication of complex shapes with high resolution.

## 1 Introduction

Thermosets are known for their exceptional properties, including excellent thermal and chemical resistance and outstanding mechanical performance.^1^ These characteristics stem from their covalent three-dimensional cross-linked network structure. However, this structural integrity poses a significant challenge in recycling, reshaping, or reprocessing this type of material, accumulating substantial polymer waste in landfills once their intended service life is concluded. A new family of thermosetting materials known as covalent adaptable networks (CANs) has been developed throughout the last 15 years. These materials behave like thermosets at service temperatures, demonstrating good mechanical performance, but they can flow like melted glass at high temperatures without losing their structural integrity. This feature enables their recycling, reprocessing, or reshaping, resembling the behavior of thermoplastics. To achieve this, CANs contain exchangeable bonds in their structure that allow shape change due to reversible chemical processes triggered by external stimuli, such as ultraviolet (UV) light or heat.^2−4^ Therefore, CANs are suitable candidates to address the drawbacks of common thermosets. Depending on their exchange mechanism, CANs can be divided into two groups. On the one hand, dissociative CANs generally experience a sudden drop in viscosity due to the loss of their network integrity, which is caused by an initial breakage of a dynamic bond and a later formation of a new one in the same or another place. On the other hand, in associative-type CANs, the mechanism is concerted, and the viscosity gradually decreases with the temperature following an Arrhenius-type dependence. Associative CANs are also called vitrimers due to their similar behavior to the vitreous silica at high temperatures and were first reported by Leibler and co-workers in 2011.^5^ Some examples of organic reactions that have been reported as potential dynamic exchanges in vitrimers are transesterification,^6,7^ transamination of vinylogous urethanes,^8,9^ olefin metathesis,^10,11^ or disulfide exchange,^12,13^ among many others.

**Scheme 1:**
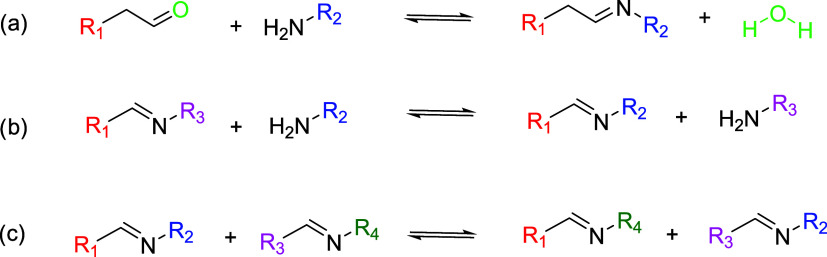
Types of Reversible Imine Reactions (a) Imine Formation/Hydrolysis, (b) Amine-Imine Exchange, and (c) Imine Metathesis

Nowadays, one of the most relevant reactions used in the vitrimer field involves imine groups or Schiff bases due to their ability to exchange groups rapidly at low temperatures, even without the presence of a catalyst.^14,15^ Imines are synthesized through the condensation reaction of aldehydes or ketones with primary amines, usually helped with an acidic catalyst under relatively mild conditions. Three options have been reported regarding the reversion mechanism: Hydrolysis, amine-imine exchange, and imine metathesis. Nonetheless, only the final two paths result in the formation of vitrimeric materials, as they follow an associative-type mechanism (Scheme 1).^3,16^ Imines are nowadays an essential type of vitrimers for their wide range of applications, such as self-healing and reusable adhesives, shape memory for innovative actuator applications, and composite materials.^17^

The development of biobased materials with reversible imine groups has been gaining attention in the recent years due to the additional potential reduction in their environmental impact produced by the use of renewable resources instead of petroleum.^18−21^ To mention some examples, Liu et al.^22^ reported two different vanillin-based vitrimers with dynamic imine bonds reacted with bisphenol A diglycidyl ether (DGEBA). The main objective was to use these materials as matrices for carbon fiber-reinforced composite materials. Since imine bonds can undergo hydrolysis under acidic conditions, the polymeric part could be recycled nondestructively, allowing the recovery of the carbon fibers. Later on, Zeng’s group^23^ described the preparation of aromatic vanillin-based monomers but using an epoxidized soybean oil (ESO) as a cross-linking agent to prepare a more sustainable material. By changing the ratio of flexible ESO and the rigid monomer, various materials with different mechanical properties were prepared from soft to tough and hard. Moreover, all exhibited excellent reprocessability, weldability, reconfigurability, and programmability. Another set of imine vitrimers was synthesized by Roig et al.^24^ by cross-linking an epoxy vanillin-based monomer with three different types of Jeffamines and a triepoxide monomer to obtain homogeneous materials and good mechanical performances. These polyimine vitrimers were able to relax the initial stress in less than 10.5 min without a catalyst and could be satisfactorily recycled.

**Scheme 2:**
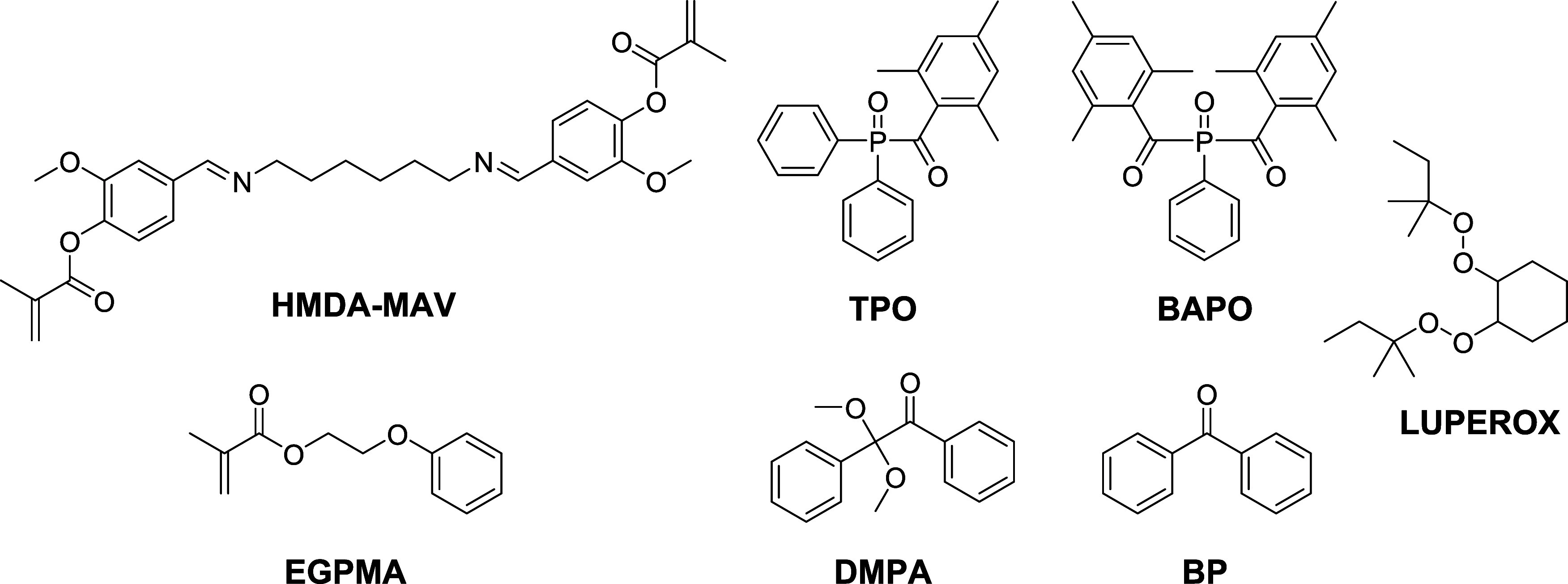
Compounds and Photo- and Thermal Radical Initiators Tested in the Preparation of the Materials

In the last decades, three-dimensional (3D) printing techniques have been applied to thermoset processing to produce objects with well-defined geometries that may be difficult to acquire using conventional techniques.^25−27^ Digital Light Processing (DLP) enables faster processing of thermoset-based printed objects than other techniques due to the layer-by-layer projection of masked images with UV light, streamlining the process and achieving pieces with better resolution in less time. Hakkarainen et al.^28^ successfully synthesized two different photocurable vanillin-based imines. The cured materials possessed good chemical and thermal resistance and relatively high *T*_g_ (above 75 °C) and could be recycled mechanically and chemically. One of the formulations was printed by DLP. Recently, Stouten et al.^29^ synthesized an acrylic photopolymer based on vanillin and dimer fatty diamine. The resulting imine vitrimers exhibited rapid relaxation times and fast recyclability, taking only 5 min at 150 °C and 40 kN. The results of the recycled materials were comparable up to 3 cycles. In addition, all the formulations were successfully printed by DLP. Taking into consideration all of the above statements, a series of vitrimeric imine materials based on vanillin have been successfully synthesized in the present work. The process involved the synthesis of a methacrylic derivative of vanillin (MAV), followed by the condensation of the remaining aldehyde with hexamethylenediamine (HMDA), leading to the formation of a diimine-dimethacrylate derivative (HMDA-DAV) (Scheme 2). Cross-linked materials with exchangeable imine moieties were obtained by curing these monomers through radical polyaddition under both thermal and photoinitiated conditions. In order to reduce the fragility of the prepared materials, customize the network structure and thermal-mechanical properties, and achieve a suitable viscosity for 3D printing, various proportions of a monofunctional methacrylate, namely ethylene glycol phenyl ether methacrylate (EGPMA) (see Scheme 2), were incorporated into the formulations. The mechanical, thermomechanical, and vitrimeric properties of the prepared materials were evaluated. The processing ability of the formulations using DLP was demonstrated. Furthermore, additional stress-relaxation tests were performed to determine the influence of the radical initiator on the dynamic behavior of the materials. The study provides a comprehensive assessment of the mechanical and vitrimeric characteristics of these materials, showcasing their potential for diverse applications.

## 2 Experimental Section

### 2.1 Materials

4-(Dimethylamino) pyridine (DMAP), hexamethylenediamine (HMDA), methacrylic anhydride (MAA), ethylene glycol phenyl ether methacrylate (EGPMA), benzophenone (BP), 2,2-dimethoxy-2-phenylacetophenone (DMPA) and phenyl bis(2,4,6-trimethylbenzoyl) phosphine oxide (BAPO) were purchased from Sigma-Aldrich. Vanillin (Van) and sodium bicarbonate (NaHCO_3_) were provided from ACROS Organics. Diphenyl (2,4,6-trimethylbenzoyl) phosphine oxide (TPO) was supplied by Ciba Specialty Chemicals Inc. 1,1-Di(*t*-amyl peroxy) cyclohexane (LUPEROX 531 M60) was kindly supplied by ARKEMA. Magnesium sulfate (MgSO_4_) was obtained from ThermoFisher Scientific, and sodium hydroxide (granulated, NaOH) and dichloromethane (DCM) were obtained from Scharlau.

### 2.2 Preparation of Vanillin Methacrylate (MAV)

The synthesis of vanillin methacrylate was carried out following a reported procedure.^30^ Vanillin (30.43 g, 20 mmol), DMAP (0.17 g, 0.14 mmol), and MAA (33.92 g, 22 mmol) were placed in a 250 mL round-bottom flask. The mixture was left for 24 h at 60 °C under magnetic stirring. After that, the mixture was diluted in 150 mL of DCM and washed with 150 mL of saturated NaHCO_3_, 150 mL of 1 M NaOH, and water. Finally, the organic phase was dried with MgSO_4_, filtered, and the solvent was eliminated in a rotary evaporator. The final product was kept in a vacuum oven at 40 °C for 24 h to render MAV as a white powder with a yield of 90%. ^1^H NMR (CDCl_3_, δ in ppm): 9.88 (s, 1H), 7.19–7.43 (m, 3H), 6.31 (t, 1H), 5.72 (t, 1H), 3.82 (s, 3H), 2.00 (t, 3H) (See Figure S1 in Supporting Information). ^13^C NMR (CDCl_3_, δ in ppm): 165.06, 159.80, 151.57, 141.90, 135.42, 135.22, 127.87, 122.80, 121.80, 110.27, 61.42, 55.89, 30.73, 27.08, 18.34 (see Figure S2).

### 2.3 Synthesis of Imine Methacrylic Monomer (HMDA-MAV)

The imine methacrylic monomer was synthesized by adapting a previously reported procedure.^30^ A mixture of MAV (10 g, 2 mmol) and HMDA (3.00 g, 1 mmol) was stirred in DCM (170 mL) for 4 h at room temperature. Then, the mixture was washed with 1 M NaOH several times and finally with water, dried over MgSO_4_, and filtered, and the solvent was eliminated in the rotary evaporator. The final product, a yellowish viscous liquid, was collected with a 70% yield. ^1^H NMR (CDCl_3_, δ in ppm): 8.10 (s, 2H), 7.30–6.90 (m, 6H), 6.20 (t, 2H), 5.58 (t, 2H), 3.70 (s, 6H), 3.44 (t, 4H), 1.90 (s, 6H), 1.60 (t, 4H), 1.29 (t, 4H) (See Figure S3). ^13^C NMR (CDCl_3_, δ in ppm): 191.14, 164.81, 152.14, 145.26, 135.17, 127.94, 124.72, 123.50, 110.91, 56.12, 18.35 (See Figure S4).

### 2.4 Preparation of the Samples

Formulations were prepared by mixing HMDA-MAV and EGPMA in different proportions. For the photocurable formulations, 2 phr of TPO (parts per hundred parts of resin) were added. The liquid formulations were injected into rectangular molds made with two glass slides subjected to metallic clamps on both sides of a Teflon spacer cut to the desired thickness and shape of 30 × 5 × 1.5 mm^3^ dimensions. The samples were irradiated for 2 min on each side in an Asiga Flash UV chamber. A UV-postcuring was performed for 15 min in a Photopol Vacuum UV oven to ensure complete curing. Finally, a thermal dark postcuring was required for 1 h at 180 °C. In the case of thermally cured samples, 2 phr of Luperox were added to the formulations, stirred for 10 min, poured in a mold with the previously described dimensions, and cured in an oven for 5 h at 130 °C, 5 h at 160 °C and postcured 2 h at 180 °C. The samples were encoded as UV/TX where UV or T stands for the curing process (photocuring or thermal curing), and X for the percentage in weight of HMDA-MAV in the formulation. Therefore, the remaining weight percentage corresponds to the amount of EGPMA. As an example, T25 refers to a thermally cured sample with a 25% weight of HMDA-MAV and 75% weight of EGPMA.

### 2.5 Characterization Techniques

^1^H and ^13^C NMR spectra were recorded on a Varian VNMR-S400 NMR spectrometer using CDCl_3_ as the solvent. All chemical shifts are quoted on the δ scale in parts per million (ppm) using the residual protonated solvent as the internal standard (^1^H NMR: CDCl_3_ = 7.26 ppm; ^13^C NMR: CDCl_3_ = 77.16 ppm). A Bruker Vertex 70FTIR spectrometer equipped with an attenuated temperature-controlled total reflection (ATR) accessory was used to analyze the cured and recycled materials. Spectra were collected at room temperature in absorbance mode with a resolution of 4 cm^–1^ and a wavelength range from 600 to 4000 cm^–1^, averaging 20 scans for each spectrum. The collected spectra were analyzed using OPUS software. Differential scanning calorimetry (DSC) analyses were carried out on a Mettler DSC3+ instrument calibrated using indium (heat flow calibration) and zinc (temperature calibration) standards. Samples of approximately 8–10 mg were placed in aluminum pans with pierced lids and analyzed under an N_2_ atmosphere with a gas flow of 50 cm^3^ min^–1^. The thermal stability of the materials was evaluated using a Mettler Toledo TGA 2 thermo-balance. Cured samples weighing around 10 mg were degraded between 30 and 600 °C at a heating rate of 10 °C min^–1^ under a N_2_ atmosphere with a flow rate of 10 cm^3^ min^–1^.

Ultraviolet–visible (UV–vis) spectra were recorded on an 8453 UV–vis spectrophotometer from Agilent Technologies using a quartz tray to check the possible competition in light absorption between the monomer and the photoinitiator. The blank was recorded by using DCM as a solvent. For the measurement of the samples, a certain amount of the photoinitiator and HMDA-MAV were weighted, dissolved in DCM, and subsequently measured (Figure S5).

The thermomechanical properties were evaluated using a DMTA Q850 (TA Instruments) equipped with a film tension clamp and the cooling system ACS 3+. Prismatic rectangular samples with dimensions of around 30 × 5 × 1.5 mm^3^ were analyzed from −15 to 150 °C at 1 Hz, at 0.1% strain and at a heating rate of 2 °C min^–1^.

The tensile stress-relaxation studies were conducted in the same equipment with the same clamp and sample’s dimensions. To evaluate the stress-relaxation behavior, the samples were first equilibrated at the desired temperature for 3 min, and a constant strain of 1% was applied for 30 min, measuring the consequent stress level as a function of time. The stress relaxation was analyzed at different temperatures using separate specimens. The stress relaxation σ(*t*) was normalized by the initial stress σ_0_. It was assumed that the stress-relaxation process can be modeled using a simple Maxwell relationship
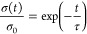
The relaxation times (τ) were determined as the time necessary to relax 0.37σ_0_. With the relaxation times obtained at each temperature, the activation energy values (*E*_a_) were calculated by using an Arrhenius-type equation
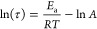
where *A* is the pre-exponential factor and *R* is the gas constant. From the Arrhenius relation, the topology freezing temperature (*T*_v_) can be obtained as the temperature at which the material reaches a viscosity η equal to 10^12^ Pa·s. For that purpose, it was assumed that τ = *E*/η and that E could be taken as the storage modulus in the rubbery state (*E*′_rubbery_) determined from DMTA (assuming *E*′ is relatively invariant in the rubbery state).

Tensile tests were conducted at room temperature using an electromechanical universal testing machine (Shimadzu AGS-X) with a 1 kN load cell at 5 mm·min^–1^ to explore the mechanical properties. Dog-bone-shaped samples (ASTM D638–14–22) (Figure S6) were tested until failure to determine the stress and strain at break and the tensile modulus of all four materials.

For the self-repairing tests, a manual press with a round steel indenter of diameter 2.5 mm was used to make plastic indentations on the sample surface by applying increasing loads of 30 kg (≈150 MPa) and 135 kg (≈400 MPa). Then, the samples remained at room temperature for 24 h to ensure no viscoelastic recovery. Subsequently, the samples were heated in an oven at 160 °C for 1 h, exploring the self-repairing process from time to time by taking pictures with a Leica Digital Microscope DMS1000 and measuring the diameter of the indentation.

Samples of the fully cured materials were ground and recycled by hot-pressing them under 5 MPa at 160 °C for 5 h in a Specac Atlas manual 15 T hydraulic hot press.

## 3 Results and Discussion

### 3.1 Study of the Curing Procedures

**Figure 1:**
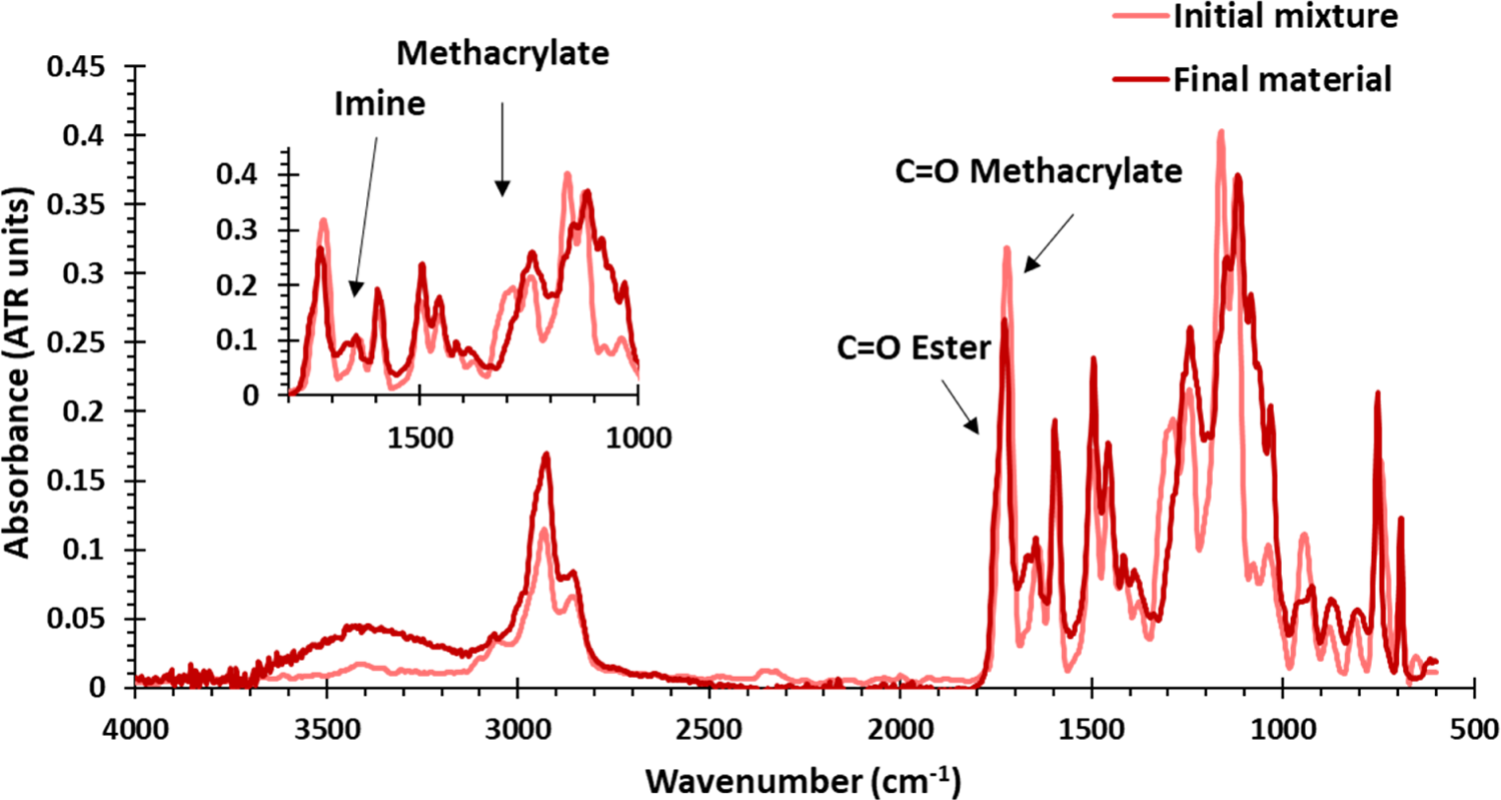
FTIR spectra of the initial mixture (pink) and the final material (red) of UV50.

Four different materials were obtained from the formulations with a content of 25 or 50% diimine-dimethacrylate (HMDA-MAV) and 75 or 50% methacrylate reactive diluent EGPMA, using a radical thermal initiator or a radical photoinitiator. EGPMA was added to reduce the cross-linking density, enhance flexibility, and improve toughness. Fourier-transform infrared spectroscopy (FTIR) was recorded on the initial formulations and the final materials to confirm that they were fully cured. Figure 1 shows the FTIR spectra before and after curing of UV50. As can be seen, the characteristic absorbance bands from methacrylates at 1350 cm^–1^ completely disappeared, and the band corresponding to the carbonyl group slightly shifts, indicating the formation of esters from the initial acrylates and confirming the successful polymerization of methacrylates. Moreover, the absorption of the imine bond at 1650 cm^–1^ remained unaltered during the whole process. All materials showed good transparency, but slight differences among them can be observed depending on the type of curing (Figure S7). Thermally cured samples presented a more intense color than their UV-cured counterparts. However, the comparison of the FTIR spectra of all the final materials reveals no significant differences in their chemical structure, as expected (Figure S8).

Finally, to evaluate the glass transition temperature (*T*_g_) and double-check the completion of the curing, a DSC analysis of the final materials was performed (Figure S9). As it can be seen, T25 and UV25 show lower *T*_g_ than their counterparts due to the lower proportion of difunctional HMDA-MAV and a higher proportion of monofunctional EGPMA. In addition, in these thermograms, it is possible to observe no residual heat evolved, which also confirms the completion of the curing.

### 3.2 Thermomechanical and Thermogravimetric Characterization of the Materials

**Figure 2:**
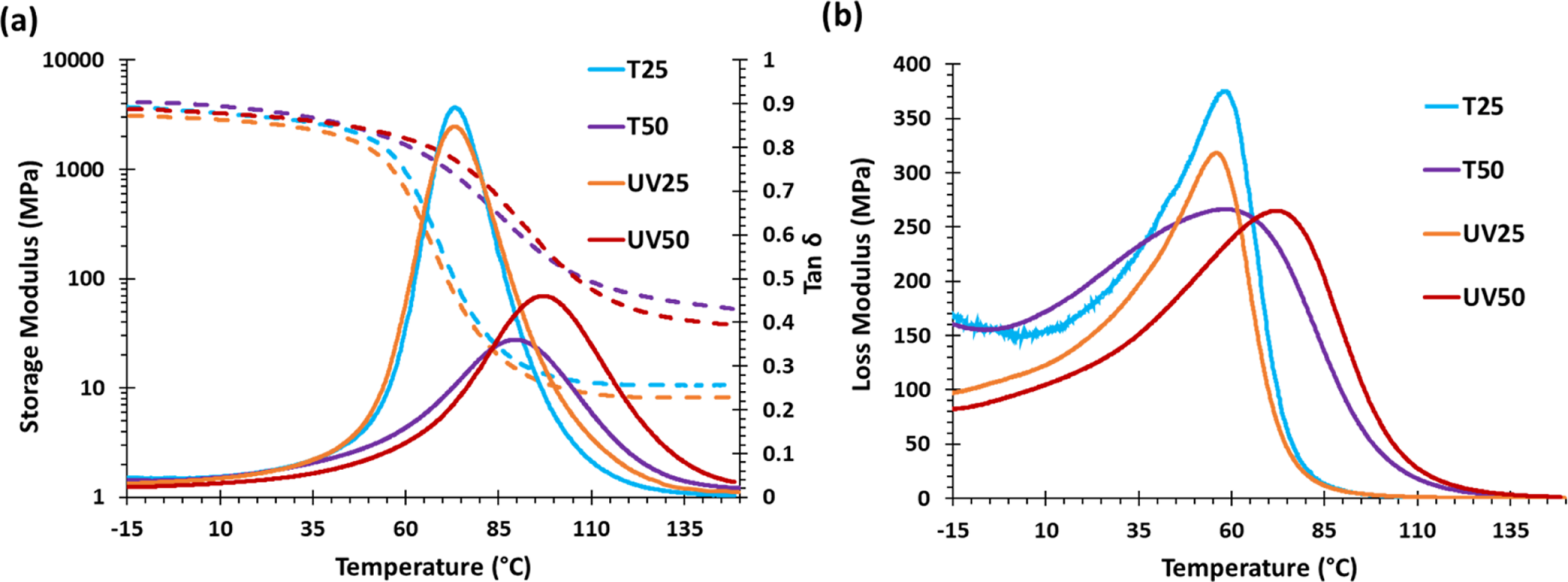
(a) Tan δ and storage modulus and (b) loss modulus evolution with temperature for all the materials.

The thermomechanical properties of all of the materials were evaluated by DMTA analysis. Figure 2 represents the evolution of the tan δ, storage (*E*′), and loss (*E*″) modulus against temperature, and the most relevant data is summarized in Table 1. As can be seen in Figure 2a, materials with 25% HMDA-MAV exhibit the same *T*_tan δ_ at 73 °C, regardless of the curing procedure. The *T*_tan δ_ values of T50 and UV50 are higher due to the increased content of the dimethacrylic HMDA-MVA, which increases the cross-linking density. However, in the UV50 photocured sample, the *T*_tan δ_ is slightly higher than that in the analogous thermal cured material. It is also possible to observe that, the higher the content of HMDA-MAV, the higher FWHM leading to more heterogeneous mixture networks as expected in a radical polymerization of methacrylates. Overall, all of these materials have *T*_tan δ_ higher than 70 °C, which ensures a glassy behavior of the materials at room temperature. The storage modulus in the glassy state (*E*′_glassy_) is very similar for all four materials, suggesting similar rigidity for all of them (see Table 1). As expected, the storage modulus in the rubbery state (*E*′_rubbery_) is higher in T50 and UV50 samples due to the higher dimethacrylate content leading to higher cross-linking density. As seen in Figure 2b, the evolution of the loss modulus of UV25 and T25 is nearly identical, while that of samples T50 and UV50 is slightly different, indicating a somewhat higher network heterogeneity in the case of the T50 sample. However, the peak of the loss modulus (*T*_E″ loss_) follows the same trend as the tan δ peak, as expected.

**Table 1:** Thermomechanical and Thermogravimetric Data for All of the Materials Prepared

sample	*E*′_glassy_^a^ (MPa)	*E*′_rubbery_^b^ (MPa)	*T*_*E*″ loss_^c^ (°C)	*T*_tan δ_^d^ (°C)	FWHM^e^ (°C)	*T*_1%_^f^ (°C)	*T*_max_^g^ (°C)	Char yield^h^ (%)
T25	2983	10.7	52	73	28	267	415	7.7
T50	2782	57.2	58	89	41	254	417	15.1
UV25	2614	8.2	56	73	25	231	415	7.6
UV50	2412	37.9	65	97	54	201	413	13.7

a Glassy storage modulus at *T*_g_ – 50 °C.

b Rubbery storage modulus at *T*_g_ + 50 °C.

c Temperature at the maximum of the peak of loss modulus.

d Temperature at the maximum of tan δ peak at 1 Hz.

e Full width at half-maximum of the tan δ peak.

f Temperature of 1% of weight loss.

g Temperature of the maximum rate of degradation.

h Char residue at 600 °C.

The thermal stability of the different materials was studied by TGA. Figure S10 shows the nonisothermal degradation curves (weight loss and derivative of weight loss). It can be seen that the shapes of the curves are similar for all materials with a main degradation peak around 415 °C, indicating that all bonds break simultaneously at this temperature. Table 1 shows that all samples lose 1% of their weight above 200 °C, indicating that safe recycling or reforming can be performed below this temperature. However, photocured materials degrade at a lower temperature than the thermally cured materials, probably due to the fact that UV25 and UV50 have a less cross-linked network in comparison to their counterparts as has been shown in *E*′_rubbery_ values. The higher the proportion of difunctional methacrylate, the earlier the onset of thermal degradation (1% weight loss temperature, *T*_1%_) and the lower the thermal stability, possibly due to the formation of volatile fragments by dynamic bond exchange reactions. The char yield of T50 and UV50 is higher due to a higher proportion of aromatic rings in the main structure of the network.

### 3.3 Mechanical Characterization

Stress–strain tests were carried out to evaluate the mechanical properties of the materials at room temperature and the effect of the composition and the curing process. Figure S11 shows the stress–strain curves, and Table 2 collects the most relevant data.

**Table 2:** Stress and Strain at Break and Tensile Modulus for All of the Materials

sample	σ_b_^a^ (MPa)	ε_b_^b^ (%)	*E*_T_^c^ (MPa)	energy absorbed at break (kJ/m^3^)^d^
T25	37 ± 1	1.3 ± 0.2	3304 ± 121	241
T50	39 ± 5	1.2 ± 0.1	3219 ± 156	234
UV25	36 ± 2	1.2 ± 0.1	2940 ± 77	216
UV50	37 ± 6	1.3 ± 0.2	3045 ± 13	241

a Stress at break.

b Strain at break.

c Tensile modulus.

d Calculated as the area under the stress–strain curve.

The tensile tests revealed that the four materials possess similar mechanical behavior at room temperature, with similar strength, rigidity, and brittle nature, as deduced from the low strain at break values. Even though T50 and UV50 are more densely cross-linked, the presence of aromatic rings from EGPMA in T25 and UV25 ensures a significant contribution from π–π interactions, leading to efficient chain packing and therefore higher stiffness.

### 3.4 Study of Vitrimeric Behavior

**Figure 3:**
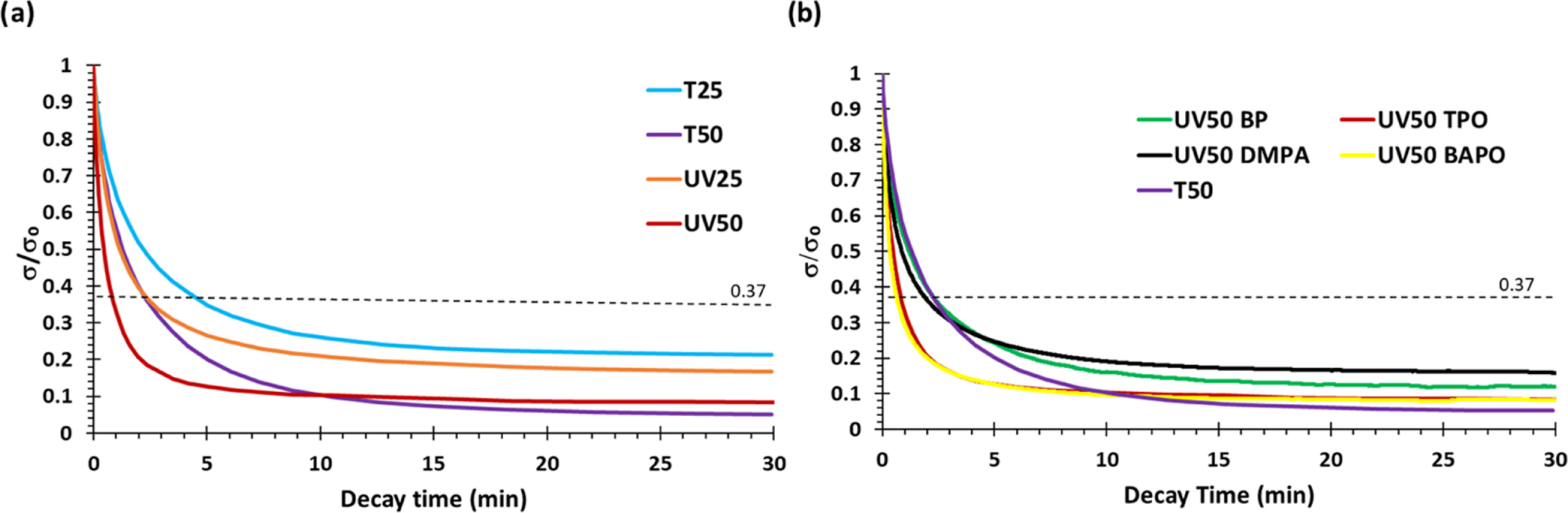
(a) Normalized stress-relaxation plot at 140 °C of all four materials. (b) Normalized stress-relaxation plots at 140 °C of the UV50 material prepared with different radical photoinitiators.

Stress-relaxation tests were carried out in DMTA in tensile mode to evaluate the vitrimeric characteristics of these materials. The stress-relaxation curves at different temperatures for all the samples are shown in Figure S12, and Table 3 presents the most important data extracted from these tests. For comparison purposes, Figure 3a shows the stress-relaxation curves at 140 °C of all of the materials.

**Table 3:** Relaxation Times, Topology Freezing Temperatures, Activation Energies, and Adjusting Parameters for the Arrhenius Equation for All Materials Prepared

sample	τ_0.37_^a^ (s)	τ_0.37_^b^ (s)	*T*_v_ (°C)	*E*_a_ (kJ/mol)	ln** A** (s)	*r*^2^
T25	276	127	27.5 ± 5.6	53.7 ± 4.2	11.45 ± 1.2	0.99
T50	129	57	51.9 ± 8.2	63.5 ± 8.5	10.43 ± 2.5	0.99
UV25	139	52	32.0 ± 11.9	66.1 ± 11.5	11.71 ± 3.3	0.99
UV50	49	34	38.4 ± 20.7	28.3 ± 7.2	10.18 ± 2.1	0.98
UV50_BP	150	-	-	-	-	-
UV50_TPO	49	-	-	-	-	-
UV50_DMPA	120	-	-	-	-	-
UV50_BAPO	37	-	-	-	-	-

a Time to reach the value of σ/σ_0_ = 0.37 at 140 °C.

b Time to reach the value of σ/σ_0_ = 0.37 at 160 °C.

The results reveal that the materials achieve the reference relaxation value of 63% (σ = 0.37σ_0_) in less than 280 s at 140 °C. The materials with a higher proportion of HMDA-MAV relax faster because of the higher imine content, despite the lower chain mobility given by the higher cross-linking density. Moreover, T50 and UV50 are able to almost completely relax the stress in less than 30 min, while T25 and UV25 only relax up to 80% with the same time and conditions due to the higher proportion of permanent bonds over dynamic bonds in comparison to T50 and UV50.

To further characterize these CANs, the time needed to relax the initial stress to e^–1^ (σ = 0.37σ_0_) at all temperatures was obtained from the corresponding relaxation curves and fitted to an Arrhenius-type eq (Figure S13). From these plots, the activation energy (*E*_a_) of the rearrangement process and the topology freezing temperature (*T*_v_) can be obtained. In all cases, the calculated *T*_v_ values are much lower than *T*_g_, evidencing that the dynamic bond exchange process is very fast. However, dynamic bond exchange is prevented by the low segmental mobility of the network at low temperatures, and therefore a sufficiently high temperature above *T*_g_ is necessary in order to observe dynamic behavior in the materials.

Nevertheless, it is worth pointing out that photocured materials relax faster than their thermally counterparts. Given that the network’s structure of the thermally cured and UV-cured materials is similar, it was hypothesized that the photoinitiator (TPO) could enhance the dynamicity by promoting the exchange reaction. To investigate it, UV50 materials were prepared using other photoinitiators, such as benzophenone (BP), 2,2-dimethoxy-2-phenylacetophenone (DMPA) and phenyl bis(2,4,6-trimethylbenzoyl) phosphine oxide (BAPO). It is worth to highlight that TPO is a phosphine oxide, and thus, BAPO was chosen as a different phosphine oxide and BP and DMPA were also tested (see Scheme 2). Figure 3b shows the stress-relaxation tests at 140 °C for the materials with different photoinitiators, and Table 3 shows the relaxation times obtained for each of them at the same temperature. As it can be observed, the materials containing DMPA and BP relaxed the stress similarly to the one obtained thermically (T50), whereas UV50-TPO and UV50-BAPO present higher relaxation rates, suggesting that phosphine oxide has a catalytic role in the imine metathesis. The fact that the materials with lower *E*′_rubbery_ (obtained from Figure S14) are not the ones that relax faster supports this hypothesis since even though their network has higher mobility, they do not relax faster than the ones that were photocured with TPO or BAPO.

### 3.5 Self-Repairing and Recycling Properties

One of the main properties of vitrimeric materials is their inherent self-repairing capabilities.^31,32^ To assess these properties, UV25 and T25 were specifically selected for testing, given that formulations with fewer imine groups pose a greater challenge for achieving successful self-repair. Controlled damage was induced in the samples through indentation using a manual press with 2.5 mm round steel indenters, applying loads of 30, 60, and 135 kg, respectively. It is worth mentioning that when applying 135 kg in T25 material, this completely broke, probably due to the sample brittleness. Subsequently, the samples were left at room temperature for 24 h to allow for any potential viscoelastic recovery processes. As the induced damage did not naturally progress at room temperature, the samples were then subjected to a temperature of 160 °C for 1 h. Remarkably, the materials exhibited complete healing during this time, as illustrated in Figure S15a for the UV25 and T25 samples.

Another important characteristic of vitrimers is their recyclability. As a proof of concept, the recyclability of these materials was assessed. The materials were mechanically ground into powder and subsequently hot-pressed under 5 MPa for 5 h at 160 °C, ensuring that the dynamic exchange reactions occurred without compromising the thermal stability of the material. Figure S15b shows the ground material and the resulting T25 and UV25 samples after recycling. To confirm the recycling procedure, DSC and FTIR analyses were performed (Figures S16 and S19), which demonstrate that no significant changes in either the chemical structure and the thermal properties occurred, suggesting a good recycling process.

### 3.6 Chemical Degradability

**Figure 4:**
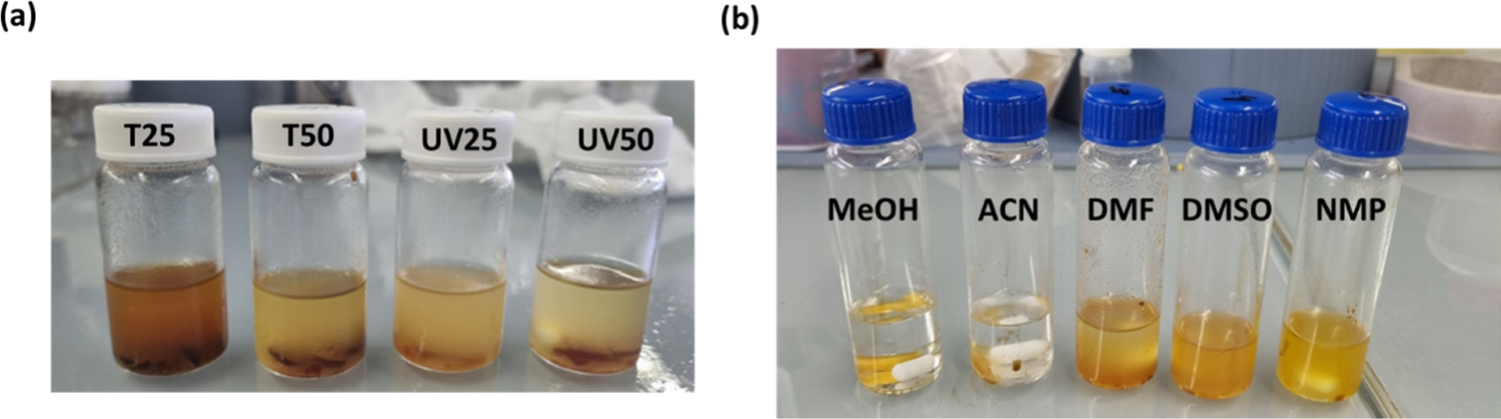
(a) Samples after chemical degradation and (b) treatment of the residue of T25 in different solvents.

Harnessing the susceptibility of imine bonds to hydrolysis under acidic conditions presents an effective means of recovering carbon fibers from their composites with polyimine matrices.^22^ To explore the degradability of the prepared materials, they were immersed in a solution of 1 M HCl/THF (1:8 v/v) for 72 h at 50 °C. As depicted in Figure 4a, the solution became yellowish over this period, and all the materials degraded up to only 77% of their initial weight. It is hypothesized that such behavior can be explained by the limited compatibility of the solvent and the hydrophobic polymer chains of the material that contain a high number of nonpolar aromatic side moieties. This may lead to inefficient swelling of the polymer backbone and therefore to limited hydrolysis of the imine groups. Moreover, even if hydrolysis was complete, solubilization of the long polymer chains produced by the radical chain-wise polymerization process would be very difficult. For this reason, the dried residue obtained after the previous step was immersed in five different polar solvents: methanol (MeOH), acetonitrile (ACN), *N*,*N*-dimethylformamide (DMF), dimethyl sulfoxide (DMSO), and *N*-methyl-2-pyrrolidone (NMP) ordered from less to more polar. After 24 h at 50 °C, the three more polar were able to completely solubilize the material, confirming the above-mentioned hypothesis (Figure 4b).

Moreover, it was also tested to combine both steps in one using a solution of 1 M HCl/solvent (1:8 v/v) for 72 h at 50 °C and as a solvent DMF, DMSO, and NMP. However, no significant differences in the sample were observed after the treatment. Even though these solvents can solubilize the previously degraded long polymer chains, they are not able to swell the initial sample.

### 3.7 3D Printing

**Figure 5:**
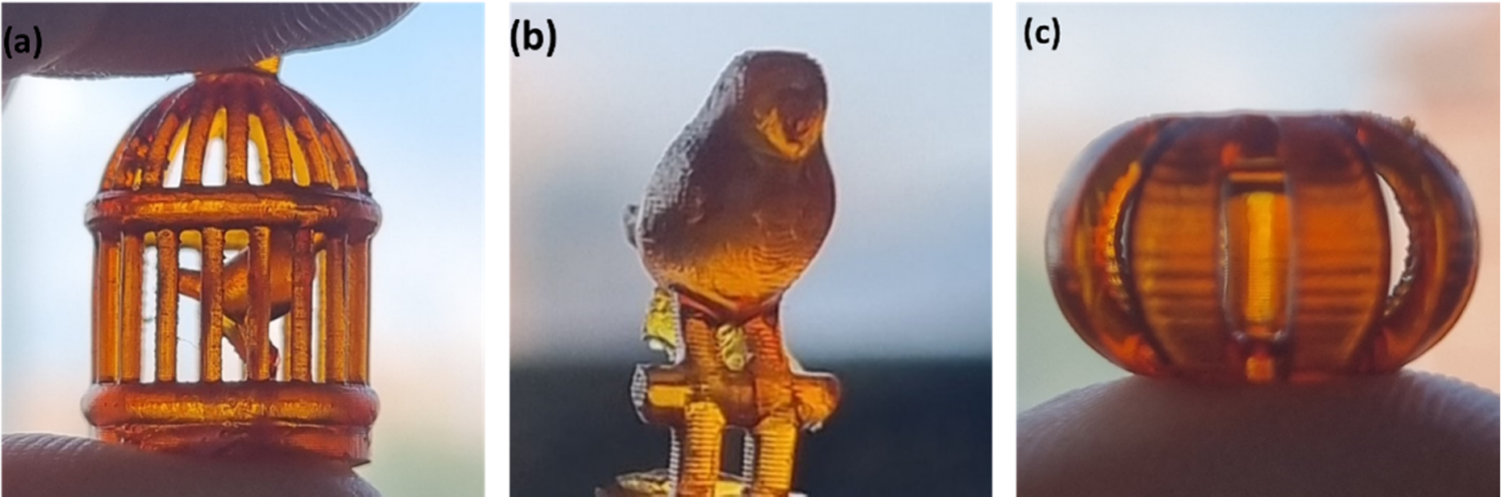
Printed objects made with UV25, (a) bird cage, (b) bird, and (c) toroid.

To investigate the applicability of the vitrimers developed in the present work for DLP-3D printing, the UV25 formulation was processed with an Asiga MAX UV DLP printer in order to create intricate objects. Objects were printed with a resolution of 100 μm in the *Z* direction. The light intensity of 8 mW/cm^2^ was used. Twelve s of UV irradiation were used for the first layer and 10 s for the subsequent layers. As illustrated in Figure 5, the complex-shaped printed items exhibited great accuracy, achieved in less than 30 min. Notably, even though they are small (around 10 mm), all the details can still be appreciated. To ensure a thorough and uniform cure, an extended UV irradiation and thermal postcuring were required. Thus, all samples underwent a 15 min treatment in a Photopol Vacuum UV oven, followed by the thermal postcuring of 1 h at 180 °C, as detailed in the experimental part.

## 4 Conclusions

Biobased imine vitrimers derived from vanillin have been successfully synthesized and characterized. Incorporating methacrylic moieties in the initial monomers enabled both thermal and photocuring processes, resulting in the obtention of cross-linked polymers. All the materials exhibited *T*_g_s exceeding 70 °C and high thermal stability (*T*_1%_ > 200 °C).

The materials prepared benefit from imine metathesis, enabling swift relaxation rates without the need for an external catalyst. Moreover, it has been noted that photocured samples exhibit faster relaxation than thermally cured ones. This acceleration is mainly attributed to the catalytic effect of phosphine oxide on the imine exchange reaction. The topology freezing temperatures (*T*_v_), calculated through Arrhenius plots, were below *T*_g_ in all cases, evidencing that the bond exchange process is fast but also that temperatures sufficiently above the glass transition temperature are necessary for effective recycling or reforming.

The use of a monofunctional methacrylate in the formulations made it possible to tune the cross-linking density and *T*_g_s of the processed materials. However, the mechanical properties determined at room (service) temperature were similar, irrespective of their composition and curing procedure, due to the efficient chain packing achieved by the presence of aromatic rings in both the diamine cross-linker and the monofunctional methacrylate.

The materials have shown noteworthy self-repairing capabilities, making it possible to recover the original surface topology after 1 h at 160 °C. In addition, they can undergo mechanical recycling without compromising their internal structural integrity, as confirmed by DSC and FTIR analyses, following a 5 h treatment at 160 °C.

Regarding chemical degradation, although the imine groups can be hydrolyzed, full solubilization of the materials could not be achieved in one step. However, by hydrolyzing the imine bonds first and subsequently dissolving the residue into a polar solvent, the samples could be completely solubilized.

Finally, the study showcased the processability of these formulations using a DLP-3D printer, rendering them particularly appealing for the production of functional, recyclable, and repairable objects in additive manufacturing applications.

## Supporting Information Available

Structural characterization for all compounds and additional thermal and thermomechanical characterization of the materials, including relevant photographs of these samples (PDF)
