# Supplementary material for: Biobased Imine Vitrimers Obtained by Photo and Thermal Curing Procedures—Promising Materials for 3D Printing
Source: ACS Appl Polym Mater. 2024 Mar 14;6(6):3364–72. doi: 10.1021/acsapm.3c03234 (PMC10964192; doi:10.1021/acsapm.3c03234)
Supplement: Supplementary file 1 — ap3c03234_si_001.pdf [file ap3c03234_si_001.pdf]

SUPPORTING INFORMATION for

Biobased Imine Vitrimers Obtained by Photo and Thermal Curing. Promising Materials for 3D Printing

Anna Vilanova-Pérez,[1] Silvia De la Flor,[2] Xavier Fernández-Francos,[3] Àngels Serra,[1] Adrià Roig[1*]

[1] Universitat Rovira i Virgili, Department of Analytical and Organic Chemistry, C/ Marcel·lí Domingo 1, Edif. N4, 43007 Tarragona, Spain

[2] Universitat Rovira i Virgili, Department of Mechanical Engineering, Av. Països Catalans 26, 43007 Tarragona, Spain

[3] Universitat Politècnica de Catalunya, Thermodynamics Laboratory ETSEIB, Av. Diagonal 647, 08028 Barcelona, Spain

Corresponding author: adria.roig@urv.cat

Table of Contents:

1. **Structural characterization of the synthesized products....................................S2**
2. **UV-Vis spectra of the monomer and photoinitiator...S4**
3. **Picture of the materials..S4**
4. **FTIR spectra of all the materials S5**
5. **DSC thermograms of all the materials..S5**
6. **Thermogravimetric curves S6**
7. **Stress-strain curves of all the materials..S6**
8. **Stress-relaxation curves of all the materials...S7**
9. **Arrhenius plots of all the materials..S7**
10. **DMTA analyses of the materials with different initiators..................................S8**
11. **Pictures of indentation and recycling procedures...S8**
12. **DSC analysis of the virgin and recycled material S9**
13. **FTIR analysis of the virgin and recycled material S10**

1. Structural characterization of the synthesized products

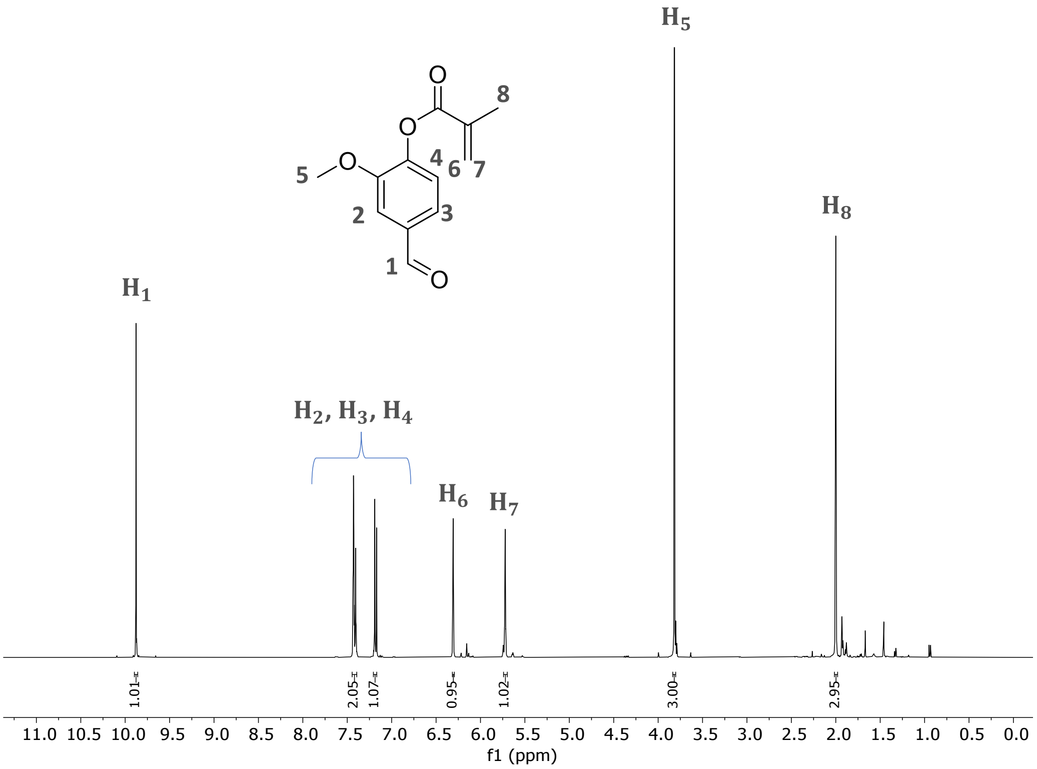

Figure S1. ^{1}H NMR spectrum of vanillin methacrylate (MAV) in $CDCl_3$.

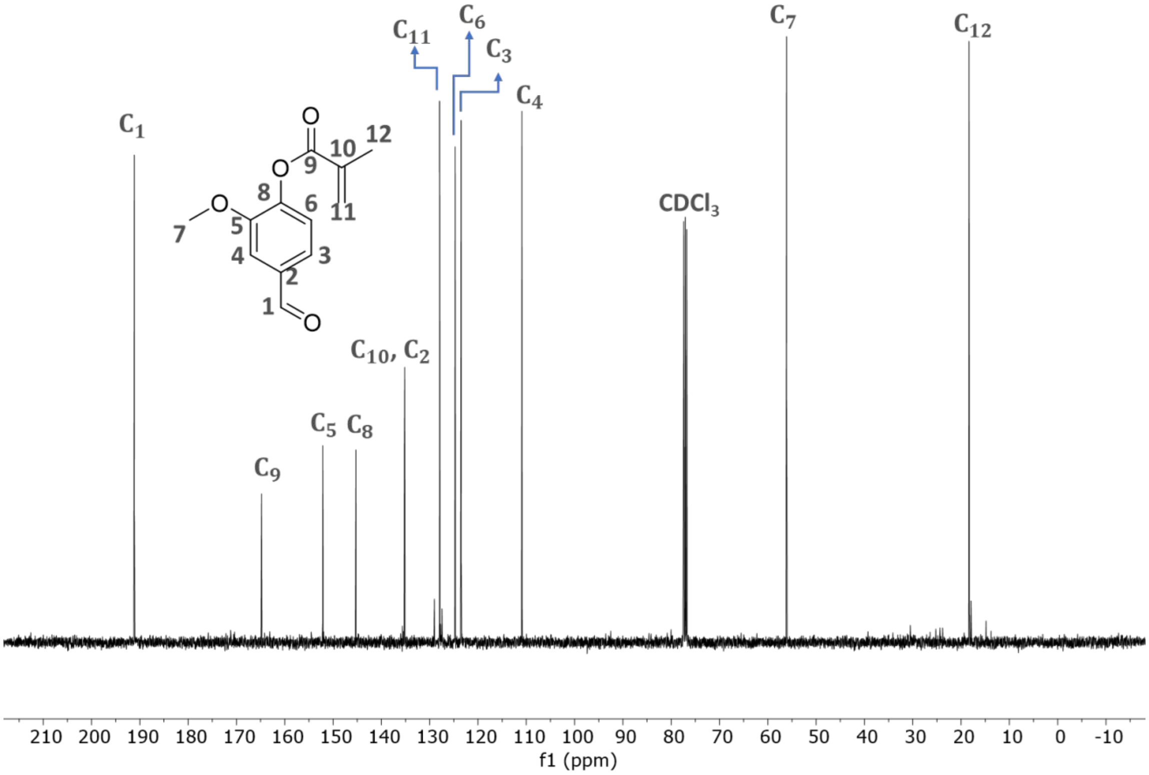

Figure S2. ^{13}C NMR spectrum of vanillin methacrylate (MAV) in $CDCl_3$.

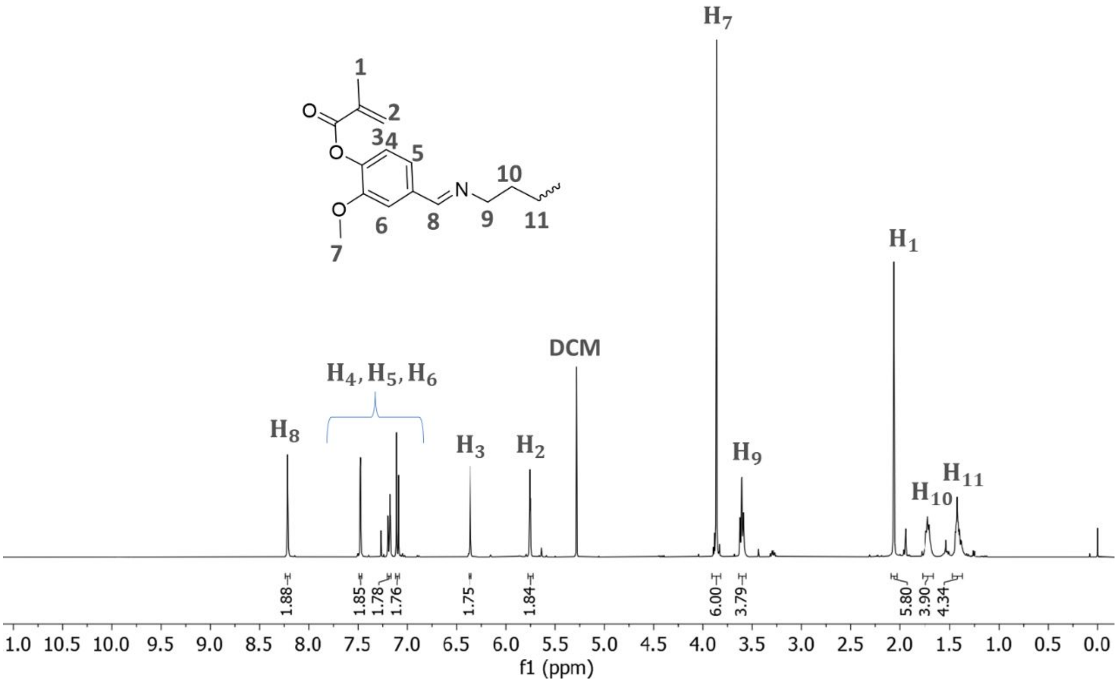

Figure S3. ^{1}H NMR spectrum of the imine methacrylic monomer (HMDA-MAV) in $CDCl_3$.

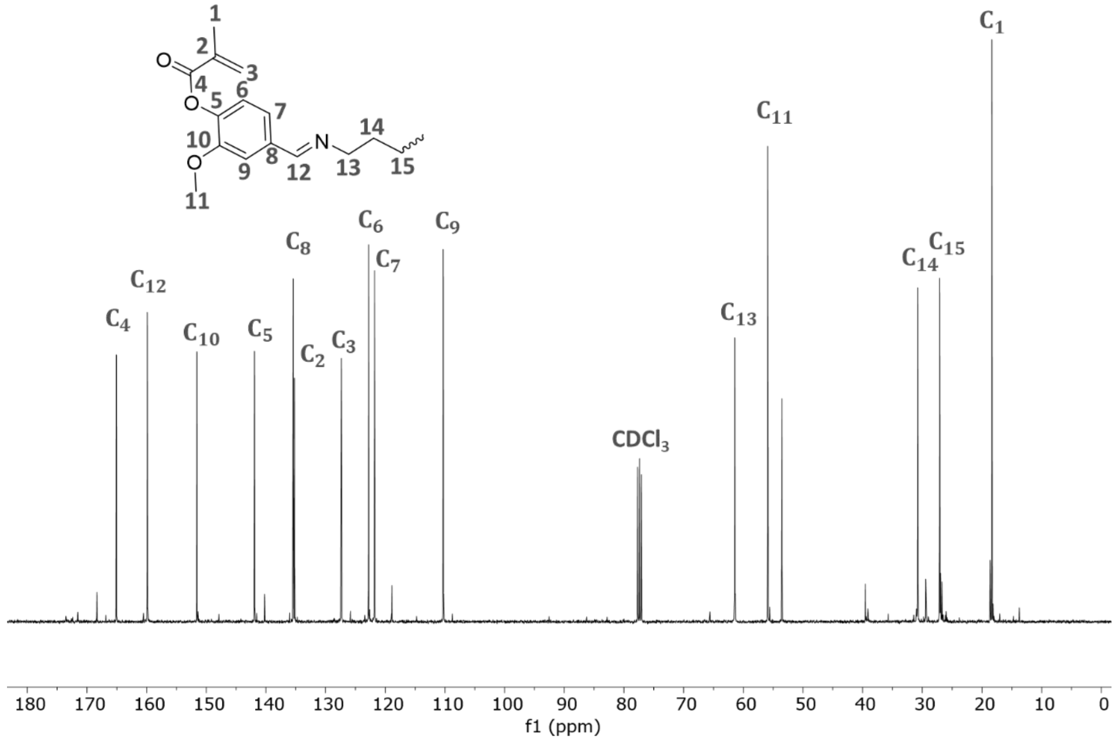

Figure S4. ^{13}C NMR spectrum of the imine methacrylic monomer (HMDA-MAV) in $CDCl_3$.

2. UV-Vis spectra of the monomer and photoinitiator

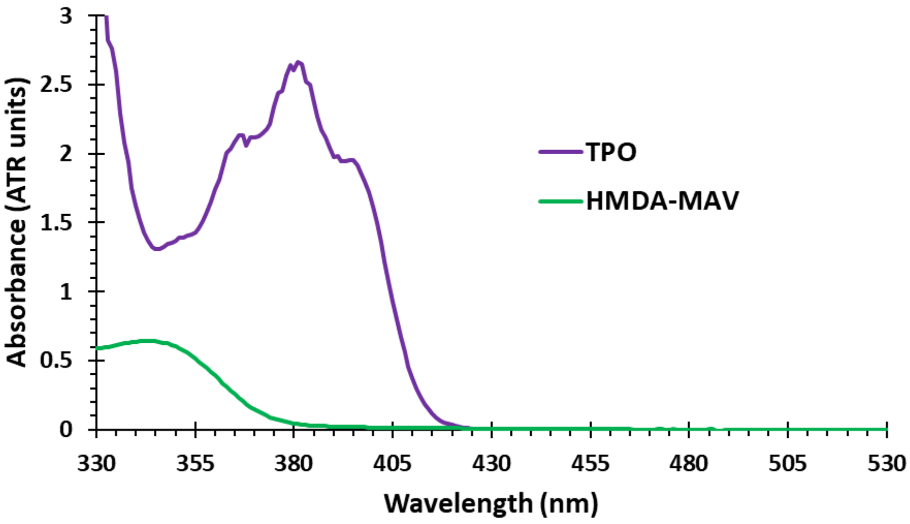

Figure S5. UV-Vis spectra of photoinitiator TPO (purple) and the synthesized monomer HMDA-MAV (green).

3. Pictures of the materials

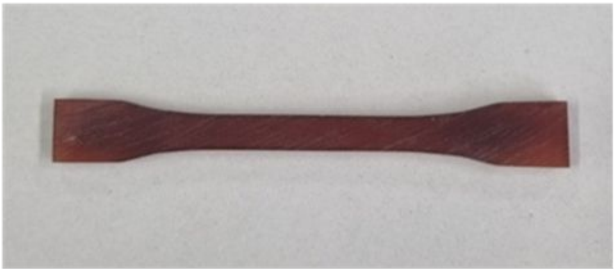

Figure S6. Example of dog-bone-shaped sample for T25.

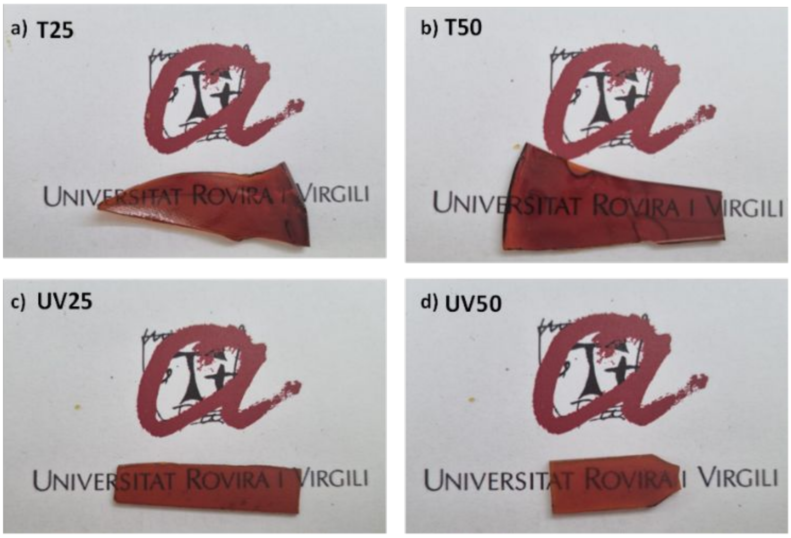

Figure S7. Pictures of reddish transparent samples of a) T25, b) T50, c) UV25 and d) UV50.

4. FTIR spectra of all the materials

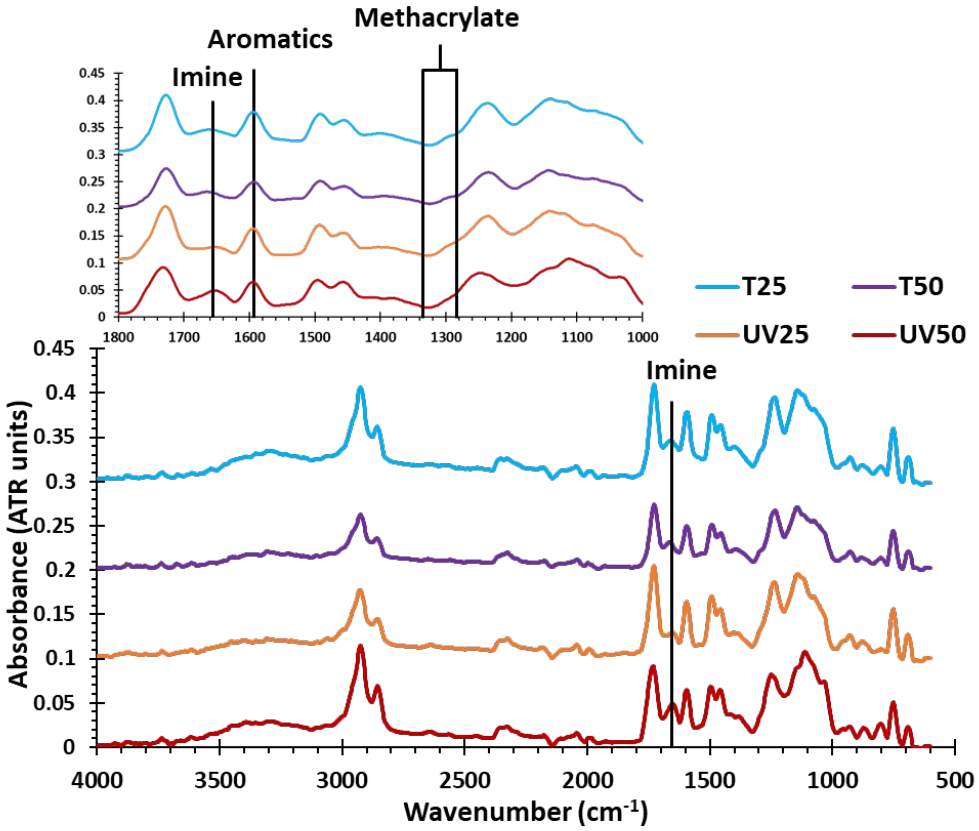

Figure S8. FTIR spectra of all the final materials.

5. DSC thermograms of all the materials

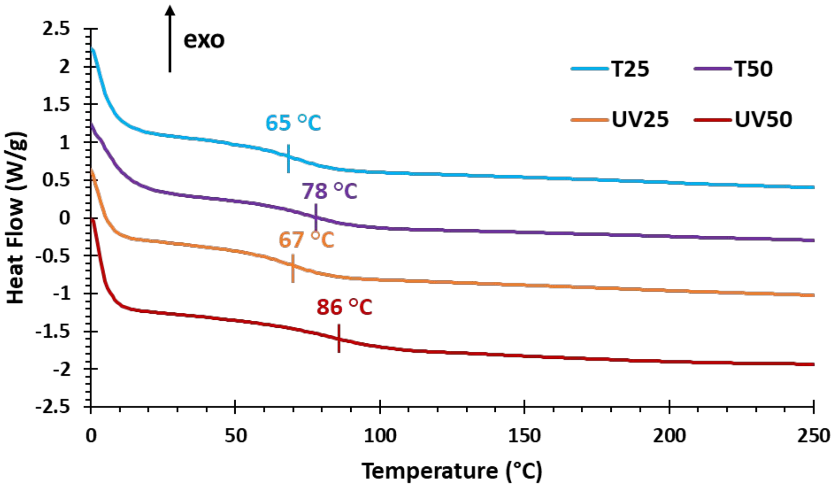

Figure S9. DSC thermograms at 50 °C·min^{-1} of all the final materials.

6. Thermogravimetric curves of all the materials

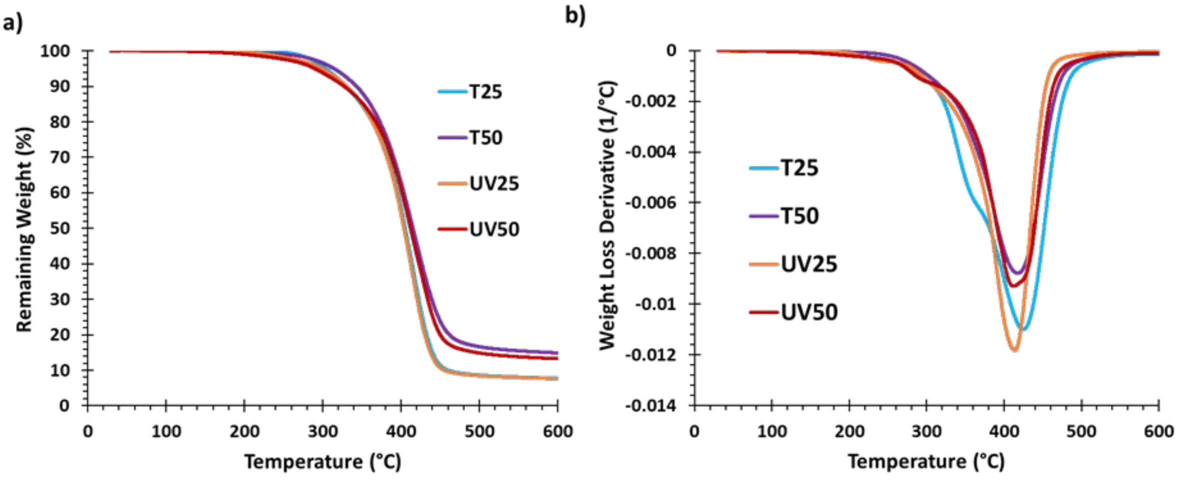

Figure S10. a) Thermogravimetric curves in N_2 atmosphere and b) DTG curves of all materials.

7. Stress-strain curves of all the materials

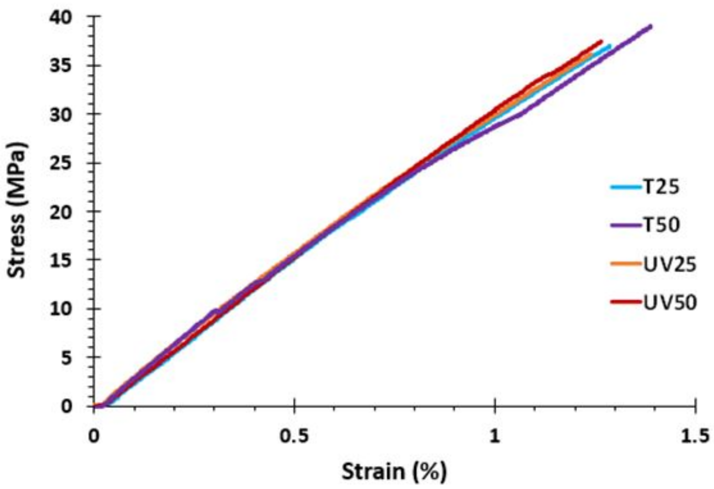

Figure S11. Tensile stress-strain curves for all the materials.

8. Stress-relaxation curves of all the materials

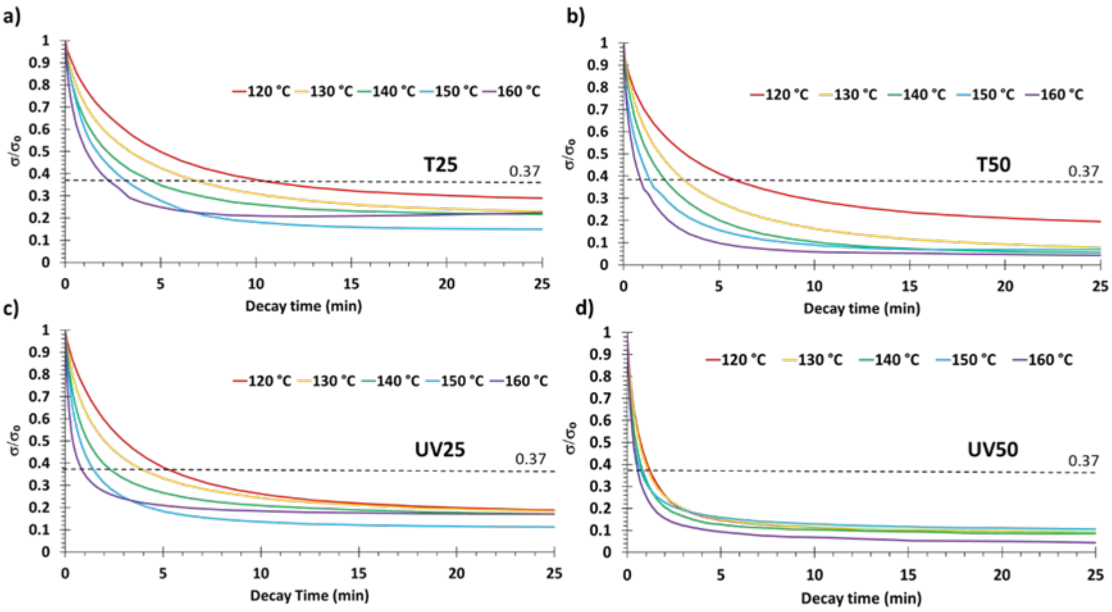

Figure S12. Relaxation curves at different temperatures for a) T25 b) T50 c) UV25 d) UV50.

9. Arrhenius plots of all the materials

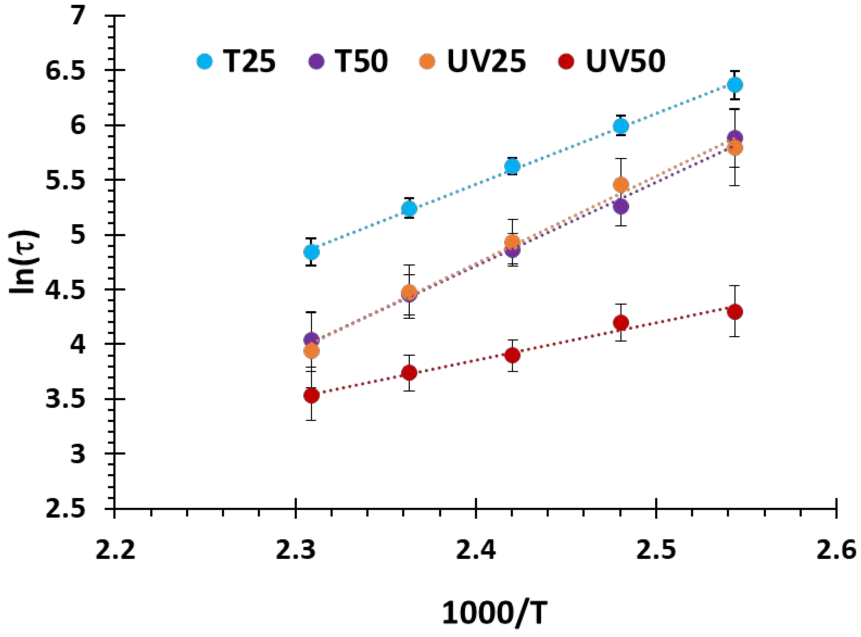

Figure S13. Arrhenius plot presenting the characteristics relaxation times against the inverse of temperature.

10. DMTA analyses of the materials with different initiators

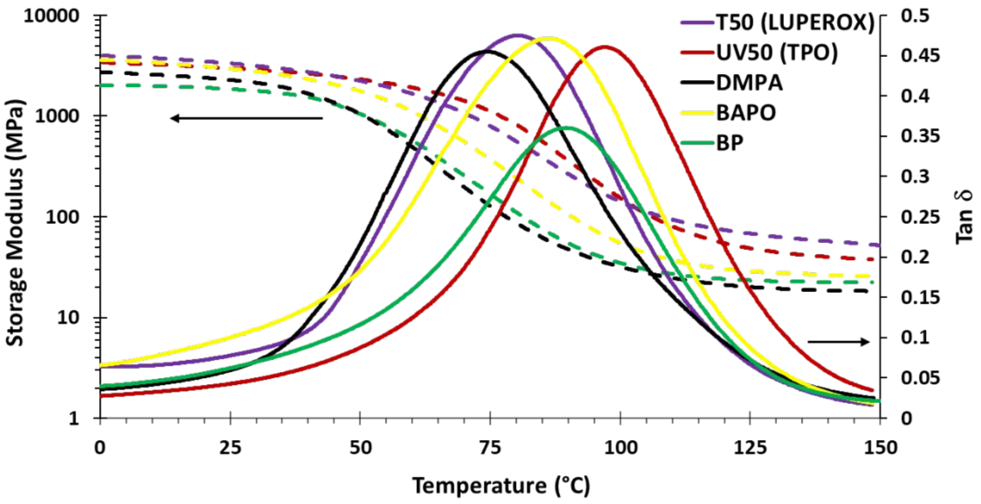

Figure S14. Evolution of *tan* δ and storage modulus with temperature for materials with different initiators.

11. Pictures of indentation and recycling procedures

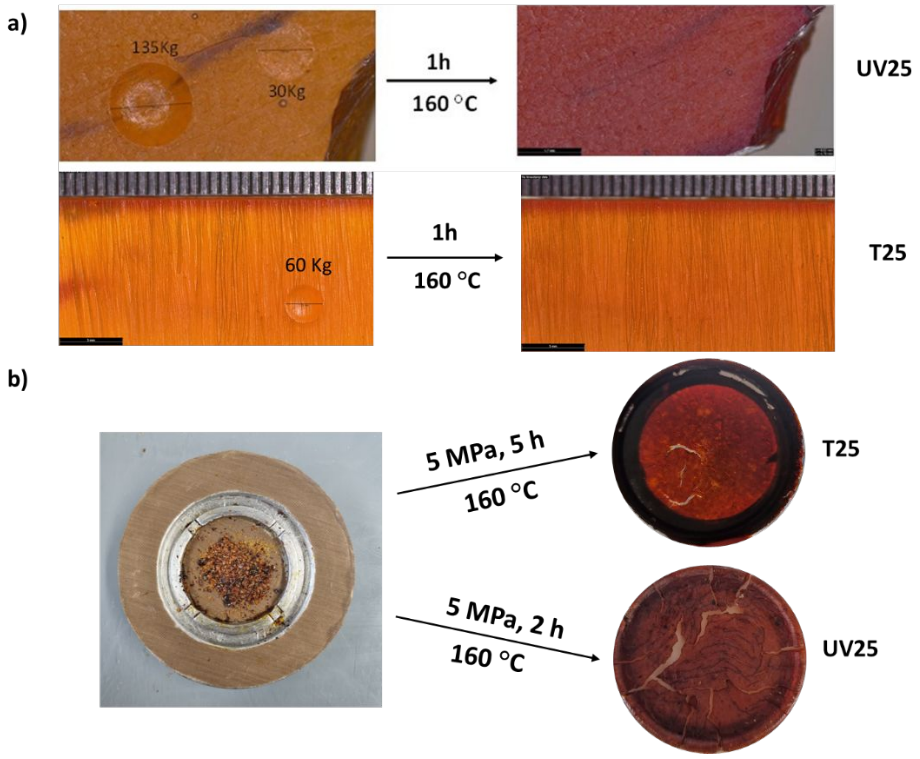

Figure S15. (a) Evolution of the indentations made to the UV25 AND T25 samples, (b) ground and recycled T25 and UV25 materials.

12. DSC analysis of the virgin and recycled material

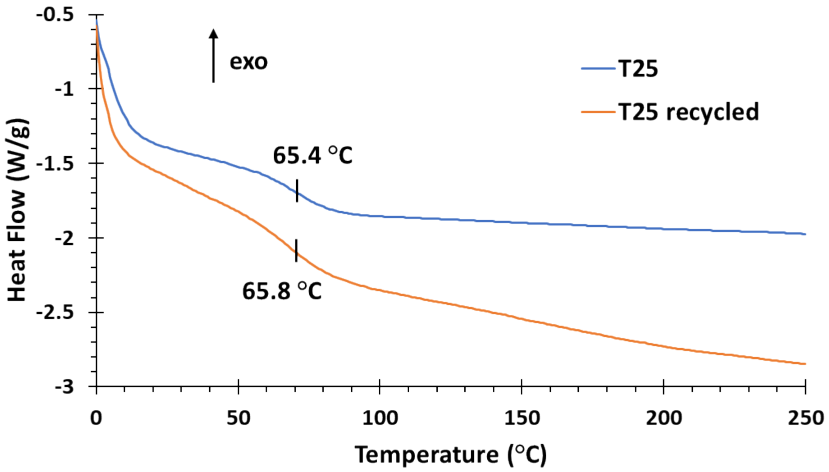

Figure S16. DSC curves at 50 °C·min^{-1} of T25 material (blue) and the recycled material (orange).

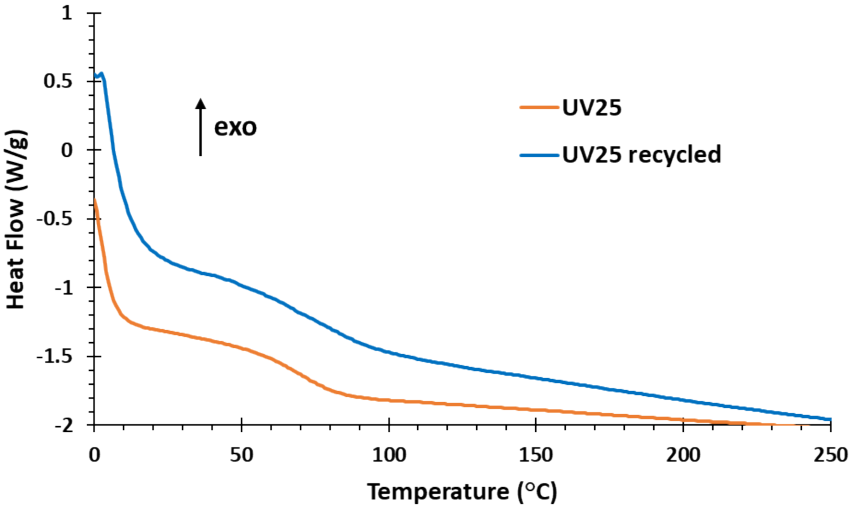

Figure S17. DSC curves at 50 °C·min^{-1} of UV25 material (orange) and the recycled material (blue).

13. FTIR analysis of the virgin and recycled material

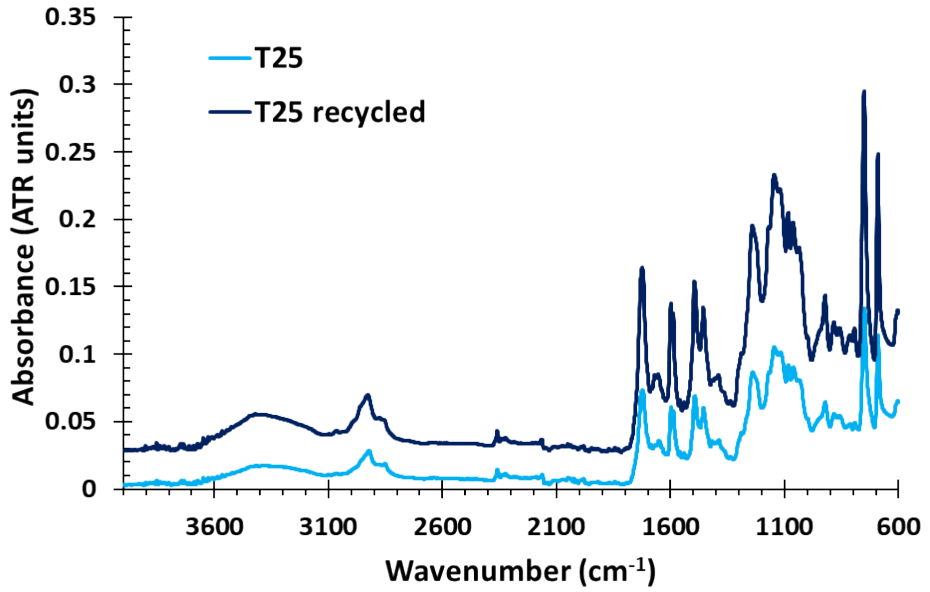

Figure S18. FTIR spectra of T25 material (blue) and the recycled material (dark blue).

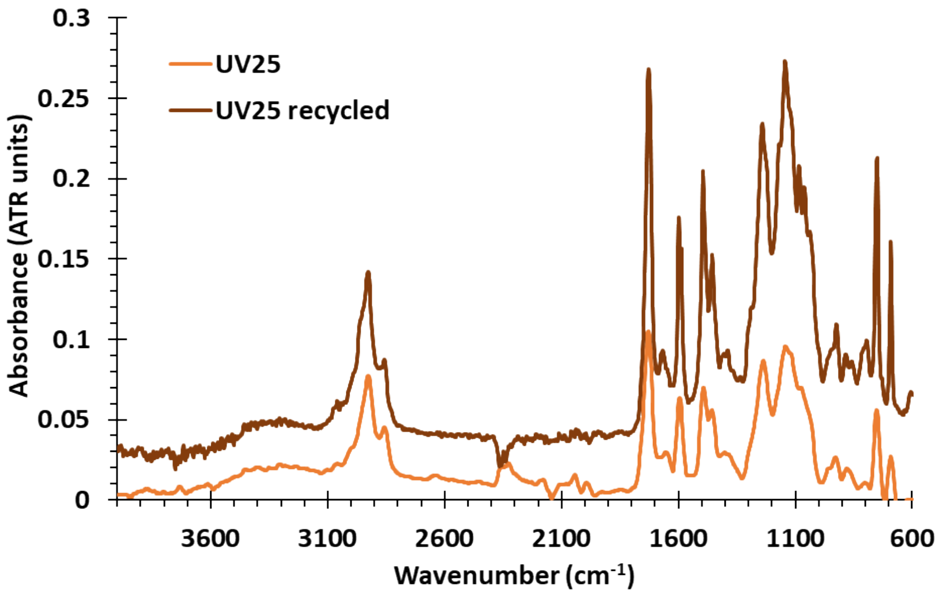

Figure S19. FTIR spectra of UV25 material (orange) and the recycled material (brown).
